# Aptamer-Mediated Electrochemical Detection of SARS-CoV-2 Nucleocapsid Protein in Saliva

**DOI:** 10.3390/bios14100471

**Published:** 2024-09-30

**Authors:** Ryan H. P. Siu, Robert G. Jesky, Yu-Jing Fan, Cyrus C. H. Au-Yeung, Andrew B. Kinghorn, Kwok-Hung Chan, Ivan Fan-Ngai Hung, Julian A. Tanner

**Affiliations:** 1 School of Biomedical Sciences, LKS Faculty of Medicine, The University of Hong Kong, Hong Kong SAR, China; u3008168@connect.hku.hk (R.H.P.S.); ryeske86@gmail.com (R.G.J.); cyrusay@connect.hku.hk (C.C.H.A.-Y.); kinghorn@hku.hk (A.B.K.); 2 Department of Medicine, School of Clinical Medicine, Li Ka Shing Faculty of Medicine, The University of Hong Kong, Pokfulam, Hong Kong SAR, China; jyjfan@connect.hku.hk (Y.-J.F.); ivanhung@hku.hk (I.F.-N.H.); 3 State Key Laboratory of Emerging Infectious Diseases, Carol Yu Centre for Infection, The University of Hong Kong, Hong Kong SAR, China; chankh2@hku.hk; 4 Department of Microbiology, School of Clinical Medicine, Li Ka Shing Faculty of Medicine, The University of Hong Kong, Pokfulam, Hong Kong SAR, China; 5 Centre for Virology, Vaccinology and Therapeutics, Hong Kong Science and Technology Park, Hong Kong SAR, China; 6 Advanced Biomedical Instrumentation Centre, Hong Kong Science Park, Hong Kong SAR, China; 7 Materials Innovation Institute for Life Sciences and Energy (MILES), HKU-SIRI, Shenzhen 518000, China

**Keywords:** electrochemical sensor, structural switching aptamer, SARS-CoV-2 nucleocapsid protein

## Abstract

Gold standard detection of SARS-CoV-2 by reverse transcription quantitative PCR (RT-qPCR) can achieve ultrasensitive viral detection down to a few RNA copies per sample. Yet, the lengthy detection and labor-intensive protocol limit its effectiveness in community screening. In view of this, a structural switching electrochemical aptamer-based biosensor (E-AB) targeting the SARS-CoV-2 nucleocapsid (N) protein was developed. Four N protein-targeting aptamers were characterized on an electrochemical cell configuration using square wave voltammetry (SWV). The sensor was investigated in an artificial saliva matrix optimizing the aptamer anchoring orientation, SWV interrogation frequency, and target incubation time. Rapid detection of the N protein was achieved within 5 min at a low nanomolar limit of detection (LOD) with high specificity. Specific N protein detection was also achieved in simulated positive saliva samples, demonstrating its feasibility for saliva-based rapid diagnosis. Further research will incorporate novel signal amplification strategies to improve sensitivity for early diagnosis.

## 1. Introduction

SARS-CoV-2 was first identified in China in 2019 and rapidly spread across the globe [1]. Despite substantial effort to control outbreaks, complete eradication has been unsuccessful because of viral genetic mutations and high contagiousness [2,3]. To this end, vaccination schemes and robust diagnostic tests remain crucial in preventing and containing further outbreaks. The globally recognized molecular diagnostic assays include RT-qPCR and lateral flow immunoassay (LFA). The PCR method sets the standard for COVID-19 diagnostic testing because of the ultrahigh sensitivity in detecting viral RNA down to a few copies per reaction by the use of sequence-specific primers [4,5]. It is the most widely accepted confirmatory test for COVID-19. However, the PCR test requires extensive laboratory equipment to carry out steps from RNA extraction to real-time quantification as well as significant manpower and time spent processing the samples and analyzing the PCR result.

LFA, on the other hand, is a paper-based optical biosensor which uses antibodies immobilized on nanoparticles and the nitrocellulose membrane to capture the viral proteins on the test strip. Major advantages include assay simplicity, portability, and efficiency. LFA can be conducted on the spot by layman within 15 min, but has inferior sensitivity compared to the PCR test. Given the drawbacks of the two common COVID-19 tests, a sensitive and rapid diagnostic assay would be advantageous.

An aptamer is a single-stranded oligonucleotide that can be folded into a unique secondary structure. This confers binding with high affinity and specificity for its target [6,7,8]. Aptamers can be chemically synthesized at low cost with diverse and flexible modification options. This makes them a promising alternative to antibodies in biosensor development. In particular, electrochemical aptamer-based biosensors (E-ABs) have promise in various diagnostic situations. By conjugating an aptamer with an electrochemical redox moiety such as methylene blue, the conformational change of the aptamer structure upon target binding can be converted to an electrochemical signal [9,10,11,12,13,14]. Electrochemical analysis has several advantages such as high signal transduction efficiency, rapid detection, potential device miniaturization, enzyme-free detection cascade, and real-time monitoring of the analyte concentration in biofluids [15,16,17,18,19,20].

To date, there are multiple studies detailing the discovery of aptamers against the SARS-CoV-2 spike (S) protein and for sensor development [10,21,22,23,24]. The spike protein is a well-known mutational hotspot, which poses challenges to identify a universal aptamer targeting existing and future variants [25]. In contrast, the N protein is more conserved to retain viral RNA packaging and replication abilities [26,27,28], which is an alternative diagnostic biomarker. Current electrochemical aptamer sensing of the N protein focuses on the use of enzymes [29,30] or free redox reporters in the electrolyte for sensitive detection [31,32]. These multi-step protocols do not favor point-of-care applications. To this end, we developed a folding-based N protein E-AB by selecting four aptamers specific to the N protein as published in the literature. The prototype is reagentless and supports single-step detection within 5 min. We describe the optimization parameters to compare and select the optimal N aptamer E-AB configuration. The E-ABs achieve a low nanomolar limit of detection with high specificity. This is also applicable in clinical saliva samples. These findings highlight the great potential of structural switching N protein E-ABs as a novel COVID-19 diagnostic platform.

## 2. Materials and Methods

### 2.1. Chemicals and Instruments

SARS-CoV-2 nucleocapsid protein was purchased from Sino Biological (Houston, TX, USA). SARS-CoV-2 spike protein (S1 domain) was purchased from GenScript (Piscataway, NJ, USA). Bovine Serum Albumin (BSA) was purchased from Affymetrix (Santa Clara, CA, USA). Artificial saliva matrix, 6-Mercapto-1-Hexanol (MCH), potassium hexacyanoferrate (III), potassium hexacyanoferrate (II) trihydrate, potassium chloride, and Tris(2-carboxyethyl) phosphine hydrochloride (TCEP) were purchased from Sigma (Saint Louis, MO, USA). PBS tablets were purchased from Thermo Scientific (Waltham, MA, USA), dissolved in MiliQ water, and filtered by 0.22 µm filter before use. DNA aptamers were synthesized from IDT (Coralville, IA, USA) with 5′ methylene blue and 3′ thiol. DNA aptamers with 5′ thiol and 3′ methylene blue were synthesized from Sangon (Shanghai, China). The DNA sequences can be found in Supplementary Information (Table S2). PalmSens4 potentiostat (PalmSens), gold disk rod working electrodes (2 mm disk diameter), platinum wire counter electrodes, and Ag/AgCl reference electrodes (in 3 M KCl) were purchased from PalmSens (Houten, the Netherlands).

### 2.2. Electrochemical Aptasensor Fabrication

Gold disk rod working electrodes were first polished sequentially by 1 μm and 0.05 μm aluminum oxide powder for 1 min each on a microcloth pad. Then, the electrodes were rinsed in absolute ethanol and water followed by water bath sonication for 10 min. The electrodes were further cleaned in 0.5 M NaOH by cyclic voltammetry over the potential range from −0.35 to −1.35 V followed by acidic scans in 0.5 M H_2_SO_4_ over the potential range from −0.3 to 1.5 V. Finally, the electrodes were scanned in 0.01 M KCl/0.1 M H_2_SO_4_ in four sequential potential ranges from 0.2 V to 1.5 V.

Thiolated- and methylene blue-modified DNA were all prepared to 100 μM in ultra-pure water. Aptamers were first reduced by TCEP in 1:100 molar ratio for 2 h in water. Then, the reduced aptamer was diluted in PBS to 1 μM. The polished and cleaned electrodes were submerged into the reduced aptamer solution overnight. After that, electrodes were washed with ultra-pure water and backfilled by 6 mM MCH in PBS for 3 h. All incubation steps were performed in RT. Finally, the electrodes were washed and stored in PBS at 4 °C before use.

### 2.3. Electrochemical Aptasensor Characterization and Measurement

All electrochemical measurements, including cyclic voltammetry (CV), electrochemical impedance spectroscopy (EIS), and square wave voltammetry (SWV) were conducted in a typical three-electrode system using PalmSens4 potentiostat controlled by PSTrace 5.9 software.

The electrochemical active surface area (ECSA) was determined by Equation (1):

ECSA = Q_Au_/400 μC cm^−2^ (1)

where Q_Au_ is the integration of charges under the reduction peak of bare gold electrode measured by CV in 0.05 M H_2_SO_4_. A total of 400 μC cm^−2^ is the estimated charge per cm^2^ of monoatomic gold oxide surface reduction. And the aptamer density was determined by Equation (2):

Aptamer density = Q_MB_/nFA (2)

where Q_MB_ is the integration of charges under the methylene blue reduction peak of aptamer-modified electrode measured by CV in 1 × PBS. n is the number of electrons transferred per redox reaction (i.e., n = 2 for MB). F is the Faraday constant. A is the electrochemical active surface area (ECSA).

The E-AB fabrication was monitored and confirmed by CV and EIS using 5 mM [Fe(CN)_6_]^3−/4−^ in PBS as the redox electrolyte. For CV, gold electrodes were scanned over the potential range from −0.3 to 0.5 V at a scan rate of 0.1 V/s. The redox oxidation and reduction peak currents from the cyclic voltammogram were automatically detected by the software. For EIS, gold electrodes were scanned in a frequency range from 1 Hz to 1 MHz with 10 mV AC amplitude and no DC potential bias. The EIS raw data were fitted to the Randles circuit to estimate the charge transfer resistance (R_ct_) values. The cyclic voltammograms from CV and the Nyquist plots from EIS are presented in Supplementary Figure S1. For protein sample detection, gold working electrodes were incubated in 1 mL of sample solution in RT for binding. Then, we interrogated the working electrode using SWV over a potential range from −0.5 to 0 V, amplitude of 25 mV, and a step size of 2 mV. Blank signals were determined in target-free buffer, and the binding signals were calculated as percentage increase or decrease relative to blank. All statistical analysis and plots were generated using Origin.

### 2.4. Nucleocapsid Protein Titration Response

N protein was diluted to various concentrations by artificial saliva. Aptamer-immobilized electrodes were first equilibrated in target-free artificial saliva matrix for 10 min before measuring the blank signal. Afterward, the sensors were washed with PBS and incubated in N protein sample solution for SWV measurement. Three electrodes immobilized with the same aptamer sequence were used for triplicate measurements. The peak methylene blue current values identified at around −0.25 V were extracted for analysis. A similar experimental approach was applied to test the sensor specificity for random sequence control.

## 3. Results and Discussion

### 3.1. Principle of Detection

**Figure 1:**
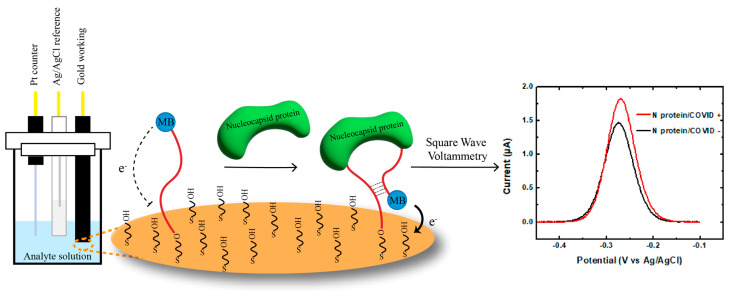
Design and mechanism of the E-AB system. SARS-CoV-2 nucleocapsid protein binding induces conformational change in the aptamers immobilized on working electrode. This results in an alteration of the distance between the MB reporter and the gold surface and leads to an increase in the current output of the system, which corresponds to the concentration of the nucleocapsid proteins in the sample.

The aptamer-mediated electrochemical sensor uses a classical three-electrode cell configuration. It consists of a platinum counter electrode, an Ag/AgCl reference electrode, and an aptamer-functionalized gold working electrode (Figure 1). The aptamers are modified with a thiol group on one end to allow the formation of an Au-S self-assemble monolayer (SAM) with MCH backfilling. Methylene blue (MB) is covalently modified on the other end of the sequence as an electrochemical redox tag. When the SARS-CoV-2 nucleocapsid protein binds to the aptamer, it induces a conformation change. The MB redox reporters come closer to the electrode surface. This increases the electron transfer rate and the current output, monitored by the square wave voltammetry technique (SWV).

In the absence of the target protein, the aptamer adopts a conformation in which the redox tag is located far away from the gold surface, resulting in a reduced electron transfer rate. However, upon binding to the N protein, the aptamer undergoes a conformational change, bringing the MB closer to the surface according to the predicted secondary structures (Table S3). This structural switching dynamic promotes the electron transfer rate, leading to an increase in the SWV signal compared to blank buffer (“Signal ON”). The change in SWV signal can be measured and correlated with the concentration of the target N protein in the sample.

### 3.2. Characterization of E-AB Fabrication

The electrochemical active surface area of the bare gold electrode was determined by the integral of the gold reduction peak (Figure S1A). It was estimated to be 0.107 ± 0.006 cm^2^ (N = 4) by Equation (1). The aptamer density was determined by the integral of the MB reduction peak and calculated based on Equation (2) (Figure S1B). The density was determined to be 1.18 ± 0.11 × 10^11^ molecules/cm^2^.

EIS and CV were performed to demonstrate the stepwise surface modification of the gold electrodes. Nyquist plots showed straight lines for all the bare gold, indicating fast electron transfer and low charge transfer resistance (Figure S2E–H, black curves). After immobilizing the DNA aptamer, semicircles appeared and indicated the increased charge transfer resistance due to the repulsion of [Fe(CN)_6_]^3−/4−^ anions with the negatively charged nucleic acid phosphate backbone (Figure S2E–H, red curves) [33]. The diameter of the semicircles reduced after MCH passivation because of the displacement of weakly bound and non-specifically adsorbed aptamers from the gold surface with the short backfilling blockers (Figure S2E–H, blue curves). The Nyquist plots confirmed successful E-AB fabrication, and the aptamers were properly orientated by MCH surface passivation. The charge transfer resistance data obtained from Randles circuit fitting are summarized in Table S1A.

The cyclic voltammograms shown in Figure S2 are consistent with the results obtained from the EIS experiments. A pair of well-defined redox peaks from [Fe(CN)_6_]^3−/4−^ was identified from the bare gold electrodes (Figure S2A–D, black curves). Upon DNA immobilization, the intensity of the redox peaks decreased due to an increase in electrode surface resistance (Figure S2A–D, red curves). The responses increased slightly after MCH passivation because of the removal of non-specifically adsorbed aptamers (Figure S2A–D, blue curves). The summary of redox oxidation and reduction current differences is provided in Table S1B.

### 3.3. Optimization of E-AB Detection Parameters

**Figure 2:**
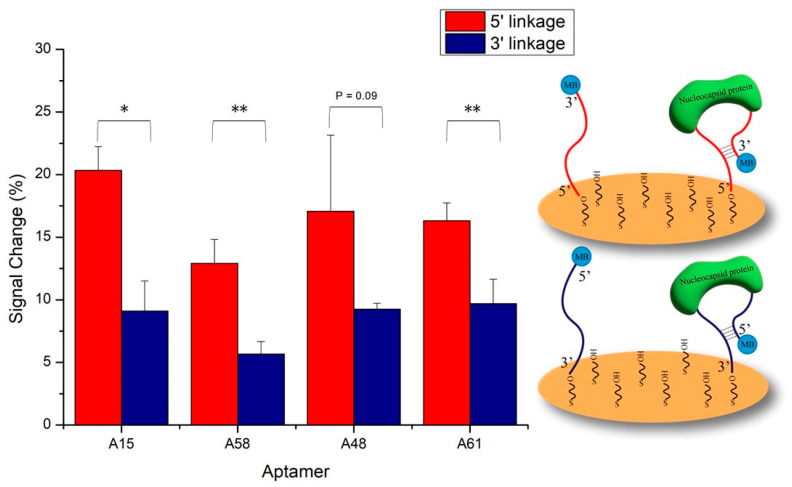
Comparison of aptamer anchoring geometry. Four selected N aptamers were decorated onto gold via either 5′ or 3′ Au-S self-assembly reaction. Blank and 50 nM target N protein in 30% artificial saliva/PBS was applied. Binding signals were measured by SWV. *, *p* < 0.05, **, *p* < 0.01.

Four N protein-specific aptamers (named A15, A58, A48, and A61) from the literature were selected to develop the E-ABs [34]. All optimizations were conducted in an artificial saliva matrix. The attachment geometry of the aptamers on gold was compared by fabricating E-ABs of each aptamer immobilized via 5′ or 3′ thiol. Since all four aptamers are predicted to share the same stem structure from the hybridization between the 5′ and 3′ ends of the sequences (Table S3), covalent conjugation from either end onto the gold is not expected to significantly alter the binding response. However, the results indicate that all the N aptamer E-ABs exhibited significantly stronger signal changes via the 5′ terminus conjugation except aptamer A48 (Figure 2). While the exact mechanism has not been elucidated, this may be explained by the aptamer’s structural flexibility due to subtle differences in the linkage orientation arising from the 5′ or 3′ hydroxyl conjugation geometry [35]. Therefore, subsequent characterization experiments were performed using an aptamer sensor prepared via 5′ thiol linkage to maximize the signal output.

**Figure 3:**
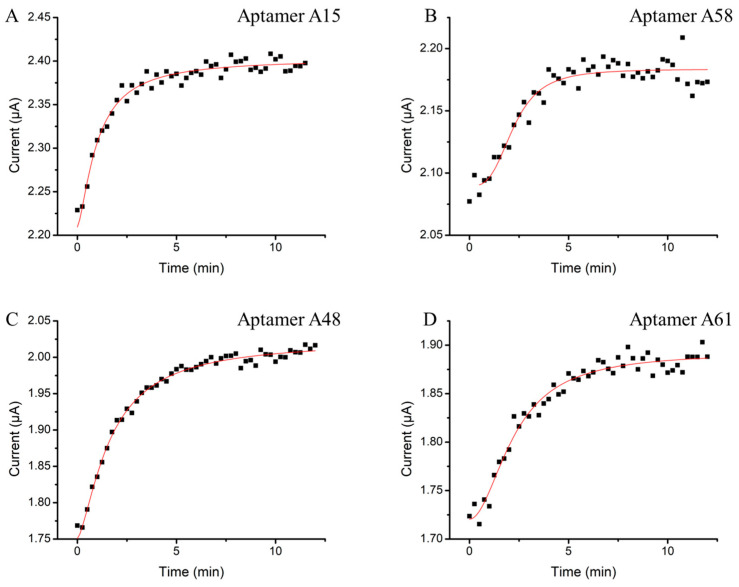
Optimization of E-AB target incubation time. Aptamers (**A**) A15, (**B**) A58, (**C**) A48, and (**D**) A61 sensors were incubated in 50 nM of N protein. The current outputs were continuously monitored from 0 to 12 min by SWV every 15 s.

To determine the minimal incubation time for signal generation, a real-time binding study was conducted. The electrochemical platform offers advantages of rapid signal transduction efficiency and the ability to achieve second to sub-second signal resolutions [20,36,37,38]. In this study, we utilized the benefits to measure the current change that originated from the methylene blue tag during the first 12 min of N protein sample incubation, with measurements taken every 15 s (Figure 3). All four N aptamers exhibited a similar binding characteristic to the N protein. The currents increased exponentially within the first 5 min of protein exposure. This indicates that the N protein is rapidly captured by the surface aptamers within this time frame. Once saturation is reached, minimal changes in current indicate that the binding has reached equilibrium and the signal has plateaued. The fast signal response from the E-ABs surpasses conventional PCR and LFA protocols which typically require at least 1 h or 15 min, respectively, before result interpretation. The rapid capturing and signaling capabilities of these aptamers on the E-AB system make it a promising candidate for future point-of-care applications.

Finally, we optimized the SWV detection frequency for the N aptamer E-ABs. SWV frequency determined the sensing behavior (signal “On” or signal “Off”) and the sensitivity of the E-ABs as demonstrated in previous folding-based E-AB research articles [9,35,39]. Additionally, signal “OFF” detection may potentially suffer from false-positive measurement from the degradation of the MB–aptamer monolayer overtime [40,41]. Therefore, an optimal SWV frequency was screened to achieve both a positive and maximized signal change. The E-AB binding signal in the three concentrations of the N protein was tested from 50 to 500 Hz (Figure S3). The N aptamer E-ABs generated a positive signal change (“On” signal) within the tested frequency range. The responses were the weakest at 50 Hz, regardless of the concentration. The signal peaked when the scans were run at 200 Hz (curve a) and remained effective when detecting the N protein at lower concentrations (curves b and c). The system specificity was retained regardless of SWV frequency when tested against non-target S protein (curve d). Overall, 200 Hz was determined to be the optimal frequency to maximize the target signal gain across target concentrations, so we decided to perform subsequent measurements at 200 Hz.

### 3.4. E-AB Sensitivity

**Figure 4:**
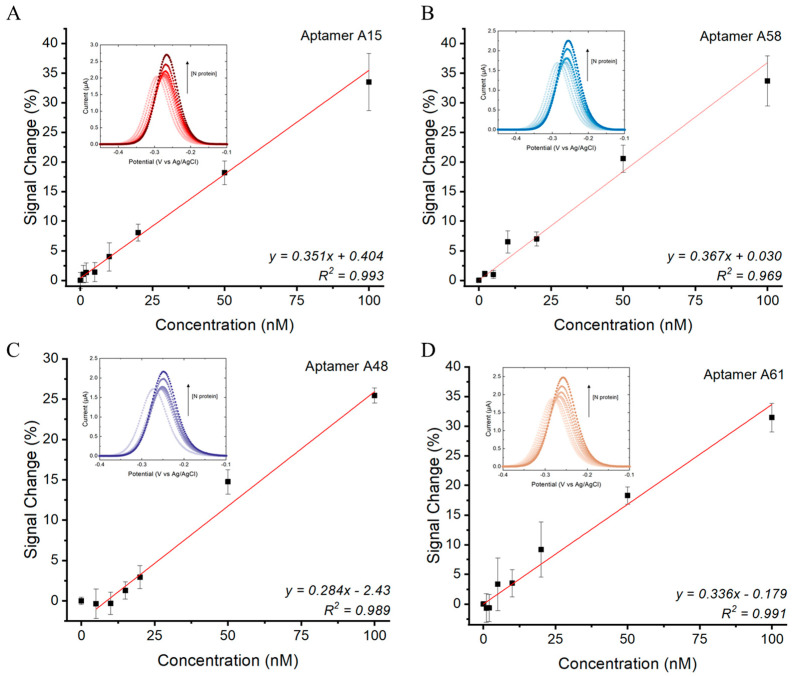
N protein concentration responses of (**A**) A15, (**B**) A58, (**C**) A48, and (**D**) A61 E-ABs. Measurements conducted using 5′ aptamer-conjugated E-ABs at 200 Hz in 30% artificial saliva/PBS. Linear regression model was used to fit the dose responses. LOD was defined as 3.3*(Sy/S), where Sy is standard error of y-intercept and S is slope of regression.

Using the optimized E-AB settings, the sensitivity of the assay was further determined in an artificial saliva matrix. Linear responses were observed for all E-ABs within the range of 5 to 100 nM of the N protein (Figure 4). The estimated limit of detection (LOD) of the A15, A58, A48, and A61 aptamer sensors toward the N protein were 2.40 nM, 1.95 nM, 10.0 nM, and 1.75 nM, respectively. The binding performance of the four aptamers was found to be comparable. This is due to the same priming sequence from the 5′ and 3′ ends shared by the aptamers (Table S2). The complementarity of the two priming ends is predicted to form an identical secondary stem structure among the four candidates (Table S3). As such, the stem structures can be stabilized by N protein binding, and the methylene blue reporters in the 3′ sequence terminal can be brought closer to the gold surface to a similar extent for all four aptamers by complementary hybridization. In addition, the order of E-AB sensitivity was found to correlate with the predicted Gibbs free energies of the aptamers in descending order (LOD/ΔG: A48 > A15 > A58 > A61). A lower ΔG is expected to result in a more stable stem secondary structure upon N protein binding. This perhaps explains why A61 exhibits the highest sensitivity due to a higher density of closely located MB to the sensor surface and vice versa for the A48 E-AB. According to the literature, this LOD range matches the N protein concentration from positive patient samples with a Ct value around 20 [42,43], indicating feasibility for clinical application.

### 3.5. E-AB Specificity and Detection of Contrived Positive Human Saliva Sample

**Figure 5:**
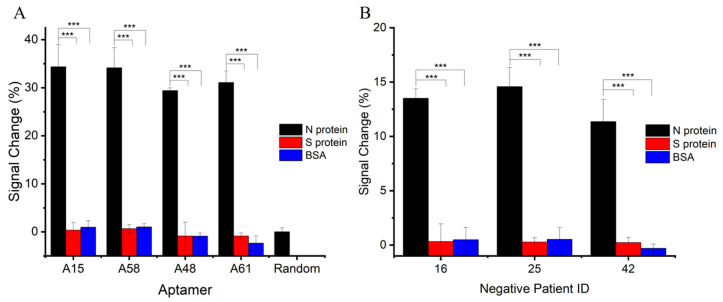
E-AB specificity in artificial saliva and human saliva matrices. (**A**) E-ABs decorated with aptamers A15, A58, A48, and A61 and random sequence were tested against 100 nM of target N protein and non-target S protein or BSA. Target protein-specific and sequence-specific detection can be concluded. (**B**) A15 E-AB was tested against target N protein and non-target S protein and BSA in three different confirmed COVID-19-negative saliva samples. ***, *p* < 0.001.

The specificity of the E-ABs in an artificial saliva matrix was assessed (Figure 5A). E-ABs binding to non-target proteins such as SARS-CoV-2 spike protein and BSA had negligible signal change responses when compared to the N protein for all aptamer sequences (*p* < 0.05). In addition, the E-AB decorated with a random sequence generated a minimal signal response to the N protein. These demonstrate the feasibility and specificity of N protein sensing by prototype.

After characterizing the E-ABs in an artificial saliva matrix, the sensor performance was verified by spiking the N protein into multiple human saliva samples using aptamer A15 E-AB as an example. Saliva samples were collected and confirmed COVID-19-negative by RT-qPCR in advance. Saliva samples were filtered through a 0.22 μm sterile filter and diluted in PBS for storage before use. N protein and non-target proteins were separately spiked into the negative samples, and the signal changes were calculated as the difference before and after. As shown in Figure 5B, significant positive signal changes were recorded for the N protein (*p* < 0.05) compared to the controls. Weaker N protein binding responses in terms of signal change percentage were recorded in the human saliva than in the artificial matrix and indicate potential interference and possibly a narrower detection range in the human sample. Further studies will need to be carried out to investigate the potential of this method in true COVID-19-positive saliva samples.

The use of aptamer conformational change for E-AB N protein sensing has not been previously reported. The current data provide insight into adapting structural switching aptamers into an E-AB format for rapid and single-step N protein detection. Proof-of-concept sensing in the human saliva matrix was also achieved. Future research will focus on verifying the sensor performance in detecting the N protein from confirmed positive saliva samples. Signal amplification strategies such as organic electrochemical transistor and upstream sample preparation are also being investigated to overcome potential interference and achieve higher sensitivity for early-stage diagnosis.

## 4. Conclusions

In this study, an electrochemical aptasensor was developed to detect SARS-CoV-2 nucleocapsid protein. Four N protein-binding aptamers were optimized for the structural switching E-AB assay. The aptamer sensors showed comparable sensitivity at a low nanomolar LOD and minimal binding to non-target proteins. The electrochemical detection method is rapid, providing results after 5 min of incubation, which is more efficient compared to conventional PCR and LFA tests. The E-AB sensing method can be applied in human saliva, which is readily available and non-invasive. This sampling method also causes less patient discomfort than nasopharyngeal and oropharyngeal swab sampling. Further research will focus on clinical sensitivity on positive saliva samples and methodologies to improve sensor performance. The described prototype could be an alternative to current COVID-19 diagnostic assays for managing future outbreaks.

## Data Availability

Dataset available on request from the authors.

## Supplementary Materials

The following supporting information can be downloaded at https://www.mdpi.com/article/10.3390/bios14100471/s1, Figure S1: Electrochemical active surface area and aptamer density characterization by cyclic voltammetry; Figure S2: Electrochemical characterization of aptasensor immobilized with aptamer A15, aptamer A58, aptamer A48, and aptamer A61 by cyclic voltammetry and electrochemical impedance spectroscopy; Figure S3: Optimization of SWV detection frequency against COVID-19 N protein and non-target S protein. Table S1: Quantitative characterization of aptamer immobilization on gold via (A) EIS and (B) CV; Table S2: SARS-CoV-2 nucleocapsid protein-specific aptamer sequences; Table S3: Predicted secondary structure and the Gibbs free energies of four N protein-binding aptamers.
