# Supplementary material for: Aptamer-Mediated Electrochemical Detection of SARS-CoV-2 Nucleocapsid Protein in Saliva
Source: Biosensors (Basel). 2024 Sep 30;14(10):471. doi: 10.3390/bios14100471 (PMC11505747; doi:10.3390/bios14100471)
Supplement: Supplementary file 1 [file biosensors-14-00471-s001.zip › biosensors-3204017-supplementary.pptx]

## Slide 1
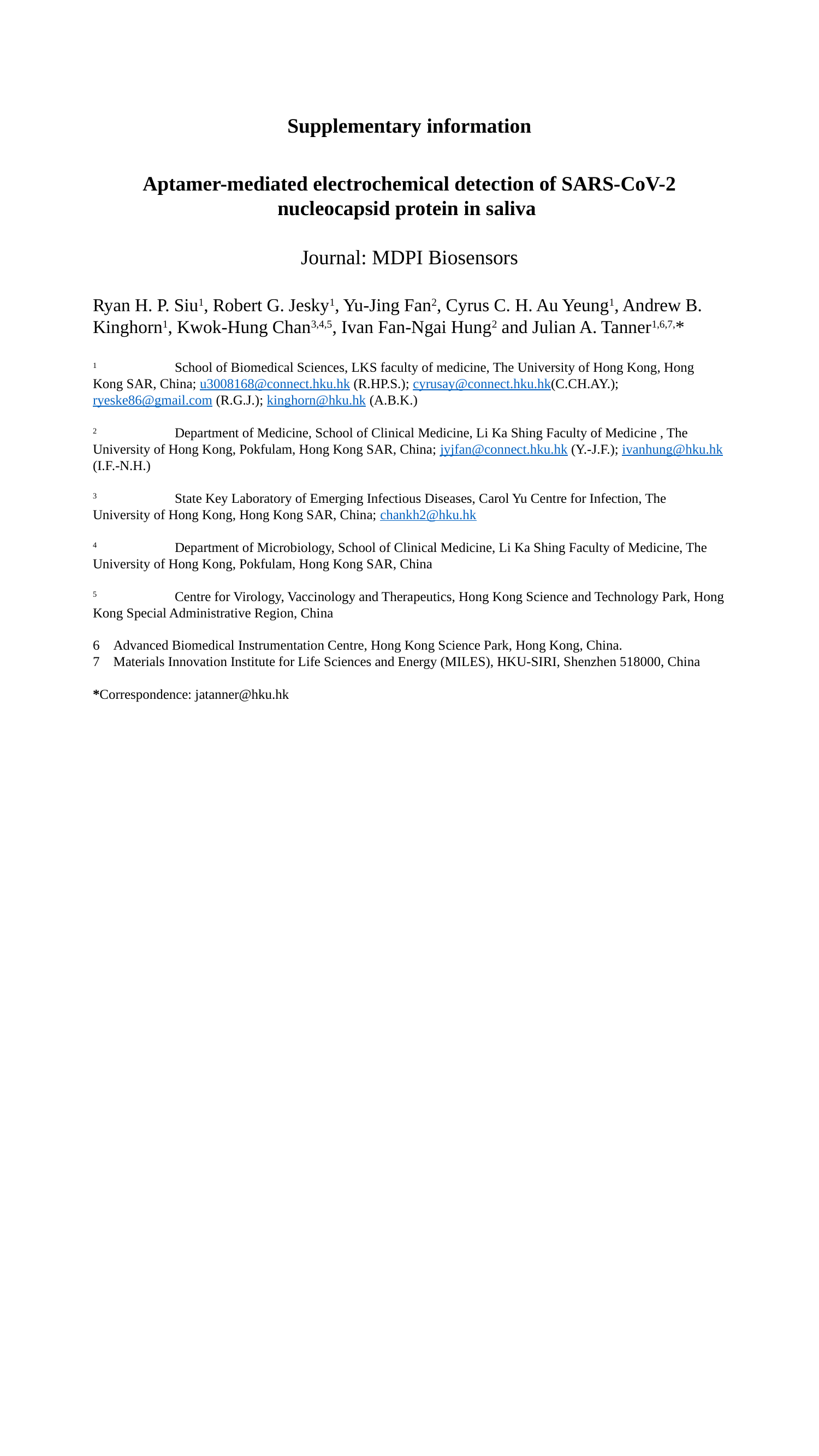

Supplementary information
Aptamer-mediated electrochemical detection of SARS-CoV-2 nucleocapsid protein in saliva
Journal: MDPI Biosensors
Ryan H. P. Siu1, Robert G. Jesky1, Yu-Jing Fan2, Cyrus C. H. Au Yeung1, Andrew B. Kinghorn1, Kwok-Hung Chan3,4,5, Ivan Fan-Ngai Hung2 and Julian A. Tanner1,6,7,*
1	School of Biomedical Sciences, LKS faculty of medicine, The University of Hong Kong, Hong Kong SAR, China; u3008168@connect.hku.hk (R.HP.S.); cyrusay@connect.hku.hk(C.CH.AY.); ryeske86@gmail.com (R.G.J.); kinghorn@hku.hk (A.B.K.)
2	Department of Medicine, School of Clinical Medicine, Li Ka Shing Faculty of Medicine , The University of Hong Kong, Pokfulam, Hong Kong SAR, China; jyjfan@connect.hku.hk (Y.-J.F.); ivanhung@hku.hk (I.F.-N.H.)
3	State Key Laboratory of Emerging Infectious Diseases, Carol Yu Centre for Infection, The University of Hong Kong, Hong Kong SAR, China; chankh2@hku.hk
4	Department of Microbiology, School of Clinical Medicine, Li Ka Shing Faculty of Medicine, The University of Hong Kong, Pokfulam, Hong Kong SAR, China
5	Centre for Virology, Vaccinology and Therapeutics, Hong Kong Science and Technology Park, Hong Kong Special Administrative Region, China
Advanced Biomedical Instrumentation Centre, Hong Kong Science Park, Hong Kong, China.
Materials Innovation Institute for Life Sciences and Energy (MILES), HKU-SIRI, Shenzhen 518000, China
*Correspondence: jatanner@hku.hk

## Slide 2
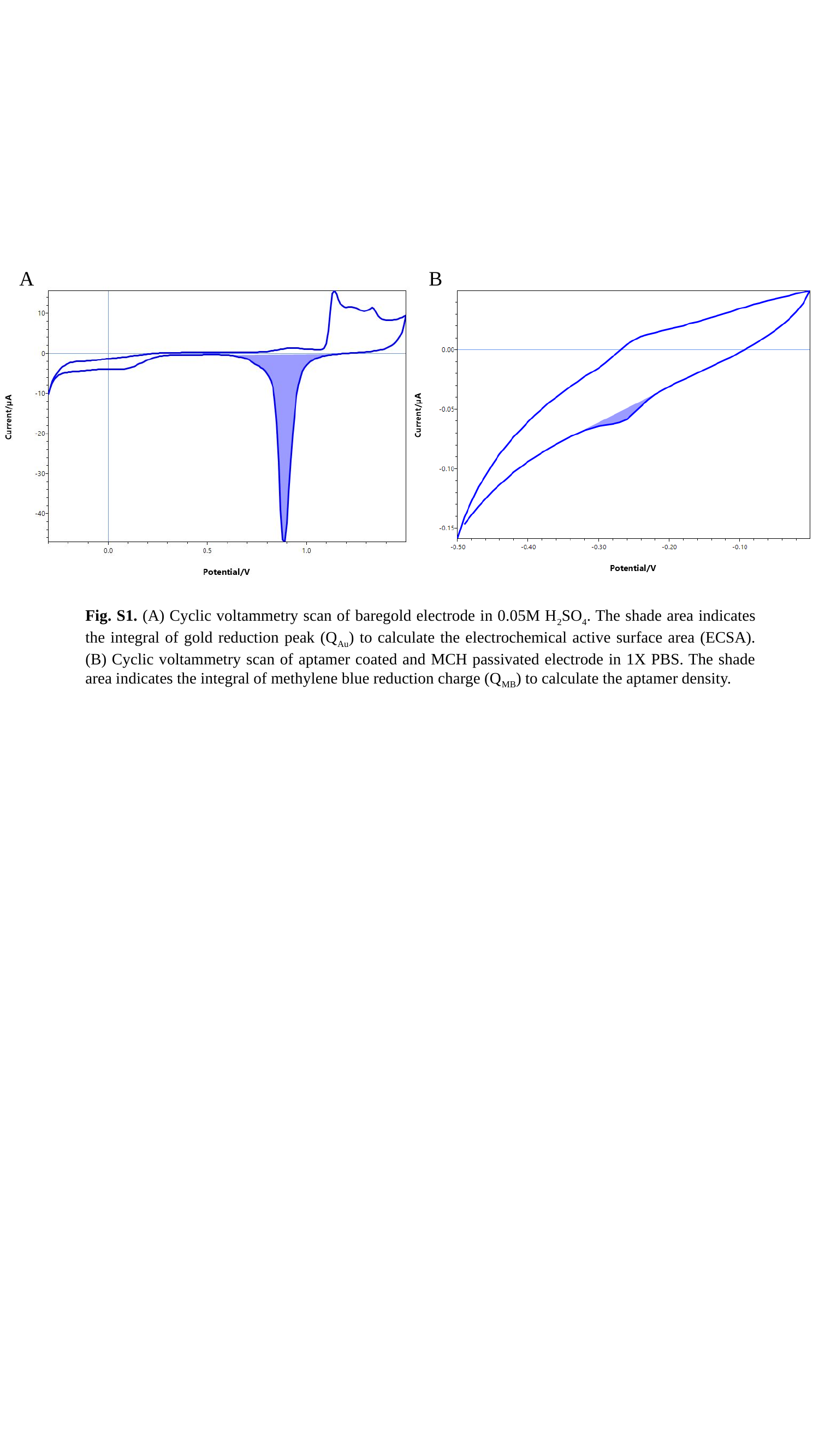

A
B
Fig. S1. (A) Cyclic voltammetry scan of baregold electrode in 0.05M H2SO4. The shade area indicates the integral of gold reduction peak (QAu) to calculate the electrochemical active surface area (ECSA). (B) Cyclic voltammetry scan of aptamer coated and MCH passivated electrode in 1X PBS. The shade area indicates the integral of methylene blue reduction charge (QMB) to calculate the aptamer density.

## Slide 3
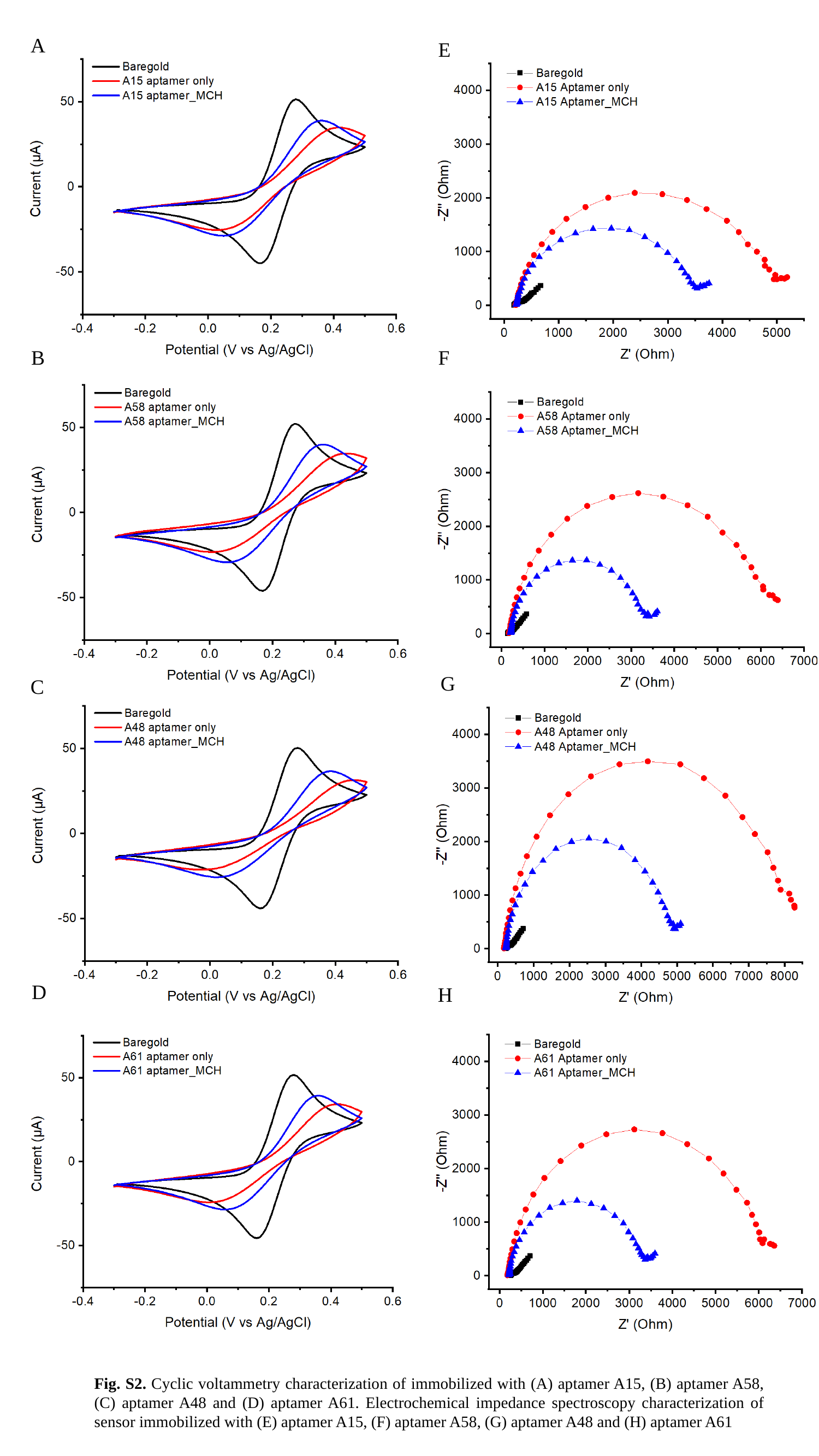

A
E
B
F
G
C
D
H
Fig. S2. Cyclic voltammetry characterization of immobilized with (A) aptamer A15, (B) aptamer A58, (C) aptamer A48 and (D) aptamer A61. Electrochemical impedance spectroscopy characterization of sensor immobilized with (E) aptamer A15, (F) aptamer A58, (G) aptamer A48 and (H) aptamer A61

## Slide 4
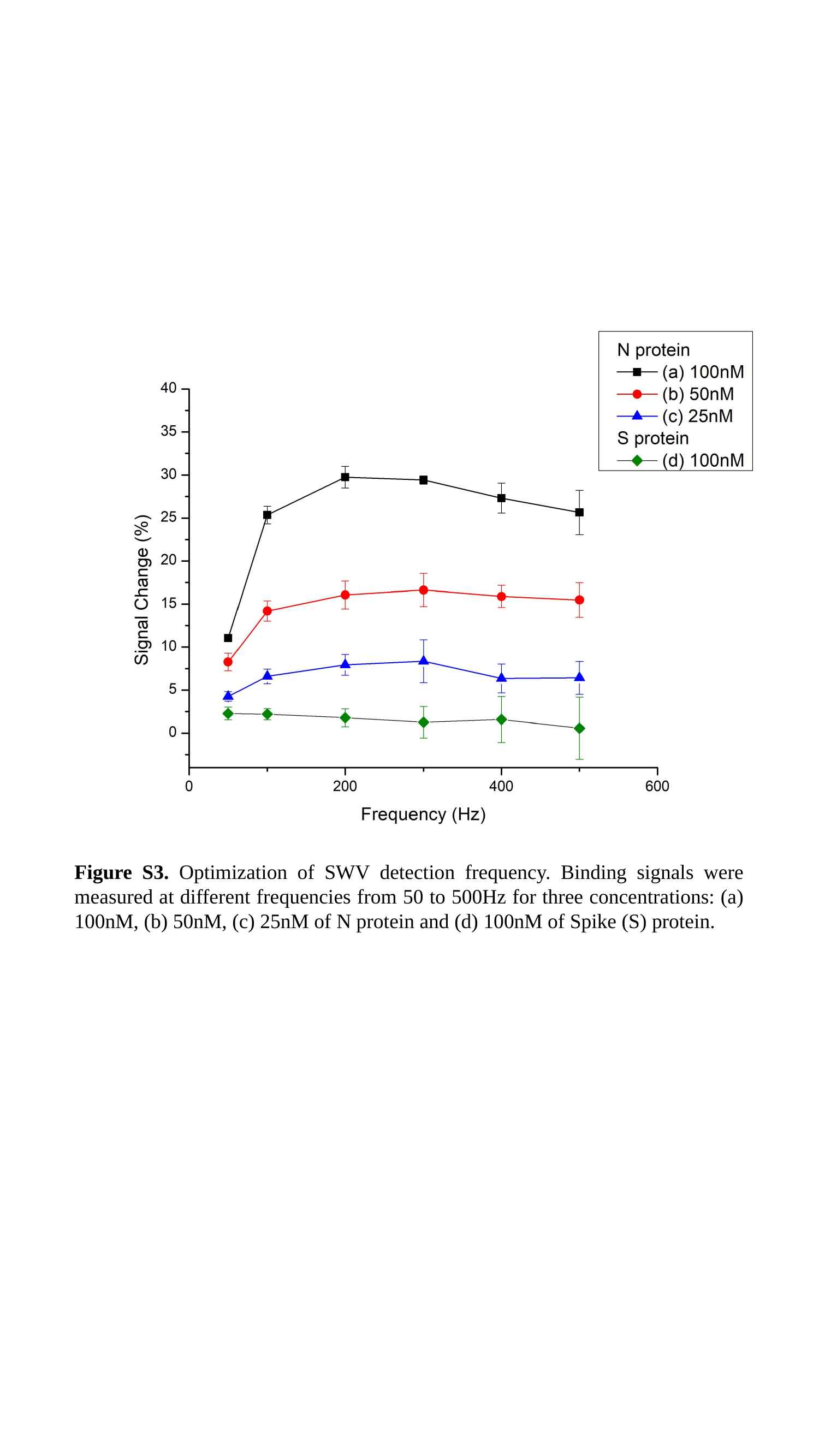

Figure S3. Optimization of SWV detection frequency. Binding signals were measured at different frequencies from 50 to 500Hz for three concentrations: (a) 100nM, (b) 50nM, (c) 25nM of N protein and (d) 100nM of Spike (S) protein.

## Slide 5
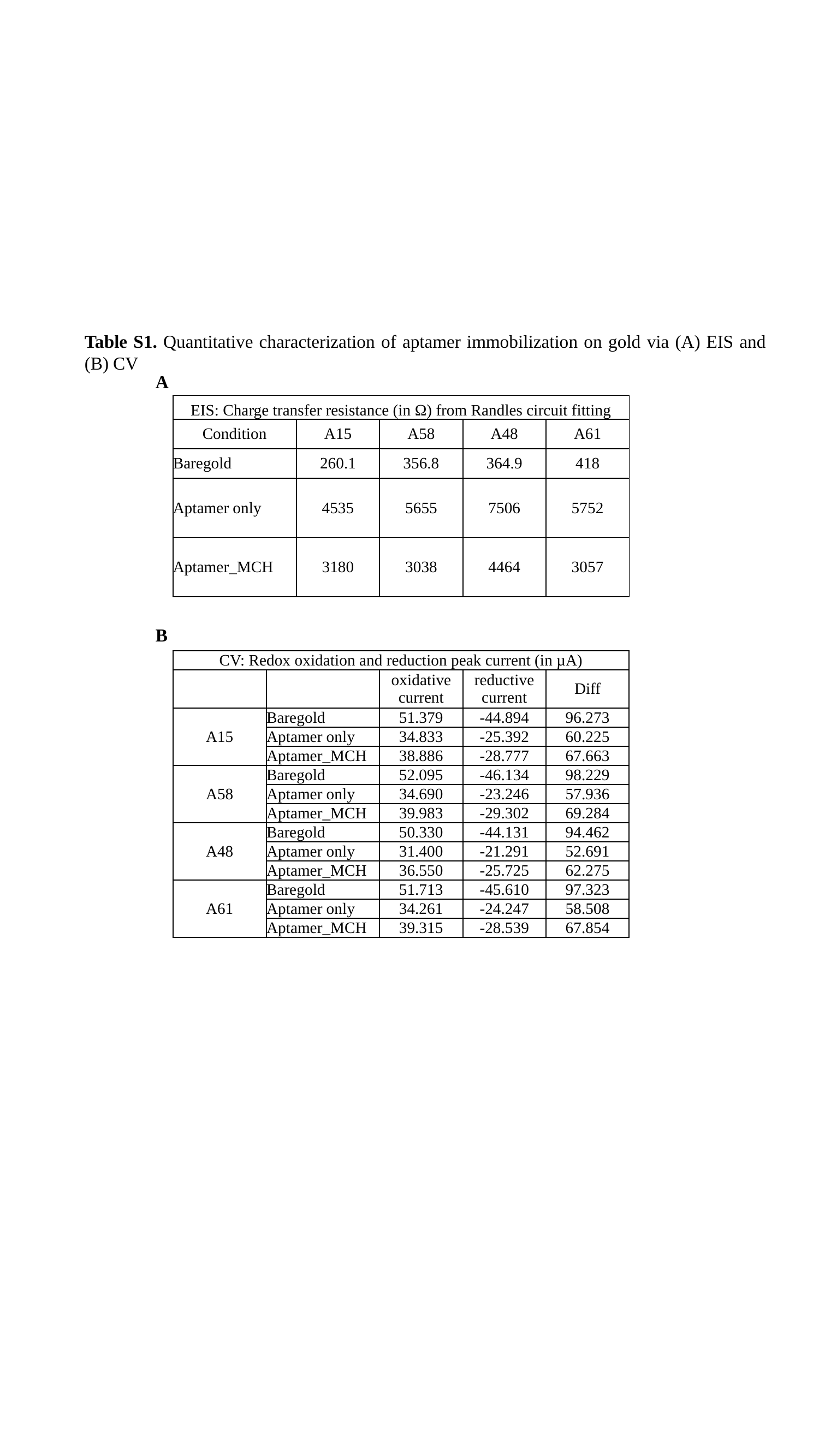

Table S1. Quantitative characterization of aptamer immobilization on gold via (A) EIS and (B) CV
A
| EIS: Charge transfer resistance (in Ω) from Randles circuit fitting | | | | |
| --- | --- | --- | --- | --- |
| Condition | A15 | A58 | A48 | A61 |
| Baregold | 260.1 | 356.8 | 364.9 | 418 |
| Aptamer only | 4535 | 5655 | 7506 | 5752 |
| Aptamer\_MCH | 3180 | 3038 | 4464 | 3057 |
B
| CV: Redox oxidation and reduction peak current (in µA) | | | | |
| --- | --- | --- | --- | --- |
| | | oxidative current | reductive current | Diff |
| A15 | Baregold | 51.379 | -44.894 | 96.273 |
| | Aptamer only | 34.833 | -25.392 | 60.225 |
| | Aptamer\_MCH | 38.886 | -28.777 | 67.663 |
| A58 | Baregold | 52.095 | -46.134 | 98.229 |
| | Aptamer only | 34.690 | -23.246 | 57.936 |
| | Aptamer\_MCH | 39.983 | -29.302 | 69.284 |
| A48 | Baregold | 50.330 | -44.131 | 94.462 |
| | Aptamer only | 31.400 | -21.291 | 52.691 |
| | Aptamer\_MCH | 36.550 | -25.725 | 62.275 |
| A61 | Baregold | 51.713 | -45.610 | 97.323 |
| | Aptamer only | 34.261 | -24.247 | 58.508 |
| | Aptamer\_MCH | 39.315 | -28.539 | 67.854 |

## Slide 6
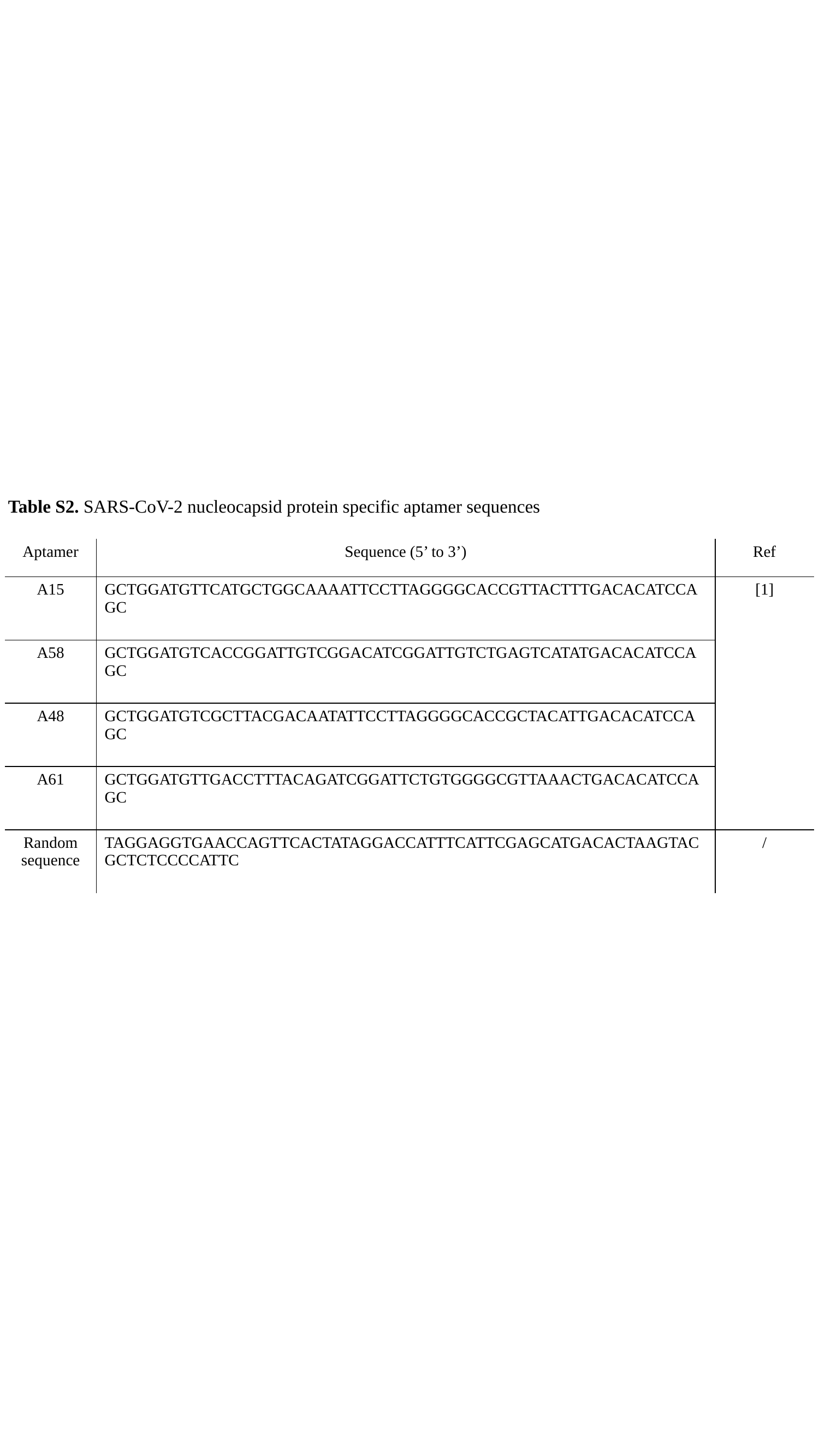

Table S2. SARS-CoV-2 nucleocapsid protein specific aptamer sequences
| Aptamer | Sequence (5’ to 3’) | Ref |
| --- | --- | --- |
| A15 | GCTGGATGTTCATGCTGGCAAAATTCCTTAGGGGCACCGTTACTTTGACACATCCAGC | [1] |
| A58 | GCTGGATGTCACCGGATTGTCGGACATCGGATTGTCTGAGTCATATGACACATCCAGC | |
| A48 | GCTGGATGTCGCTTACGACAATATTCCTTAGGGGCACCGCTACATTGACACATCCAGC | |
| A61 | GCTGGATGTTGACCTTTACAGATCGGATTCTGTGGGGCGTTAAACTGACACATCCAGC | |
| Random sequence | TAGGAGGTGAACCAGTTCACTATAGGACCATTTCATTCGAGCATGACACTAAGTACGCTCTCCCCATTC | / |

## Slide 7
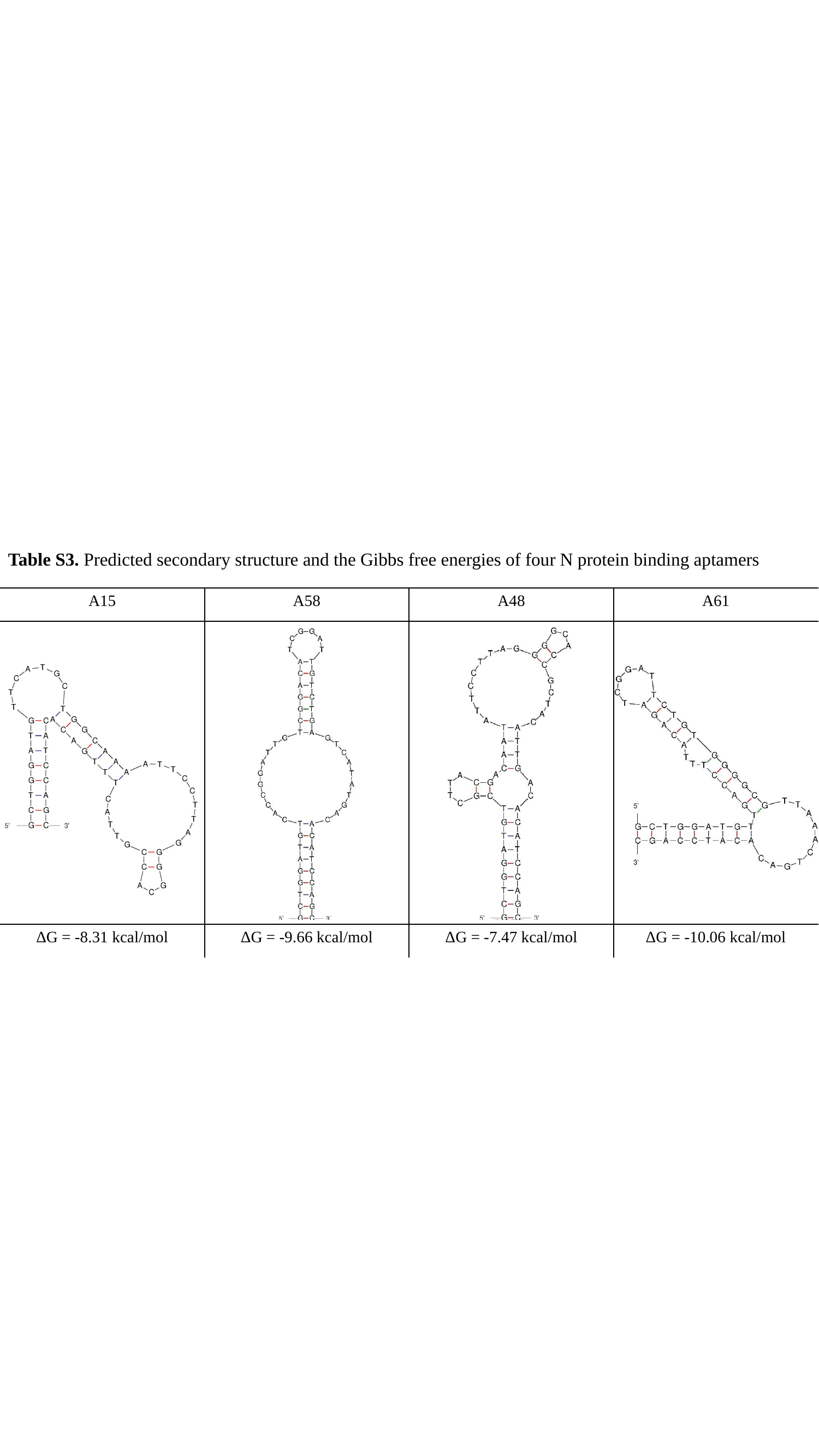

Table S3. Predicted secondary structure and the Gibbs free energies of four N protein binding aptamers
| A15 | A58 | A48 | A61 |
| --- | --- | --- | --- |
| | | | |
| ΔG = -8.31 kcal/mol | ΔG = -9.66 kcal/mol | ΔG = -7.47 kcal/mol | ΔG = -10.06 kcal/mol |

## Slide 8
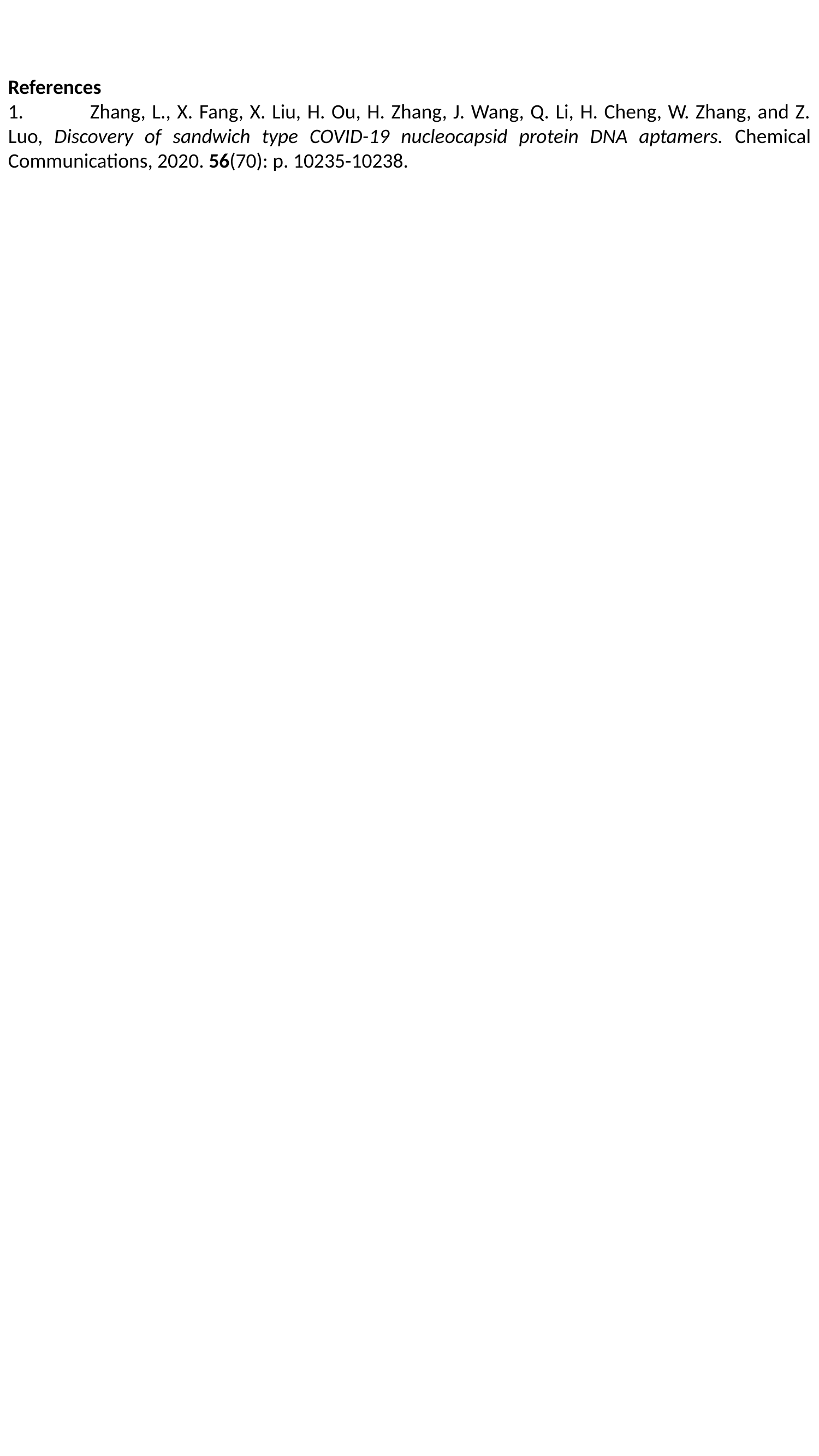

References
1.	Zhang, L., X. Fang, X. Liu, H. Ou, H. Zhang, J. Wang, Q. Li, H. Cheng, W. Zhang, and Z. Luo, Discovery of sandwich type COVID-19 nucleocapsid protein DNA aptamers. Chemical Communications, 2020. 56(70): p. 10235-10238.
